# Multi-Directional Strain Measurement in Fiber-Reinforced Plastic Based on Birefringence of Embedded Fiber Bragg Grating

**DOI:** 10.3390/s24196190

**Published:** 2024-09-24

**Authors:** Chunhua Zhou, Changhao Chen, Zilong Ye, Qi Wu, Ke Xiong

**Affiliations:** 1 State Key Laboratory of Mechanics and Control for Aerospace Structures, Nanjing University of Aeronautics and Astronautics, Nanjing 210016, China; zch777@126.com (C.Z.); chenchanghao@nuaa.edu.cn (C.C.); zlye@nuaa.edu.cn (Z.Y.); kxiong@nuaa.edu.cn (K.X.); 2 Shanghai Institute of Satellite Engineering, China Aerospace Science and Technology Corporation, Shanghai 201109, China

**Keywords:** multi-directional strain, fiber reinforced plastic, fiber Bragg grating, birefringence, strain transfer

## Abstract

Embedded fiber Bragg gratings are increasingly applied for in-situ strain measurement in fiber-reinforced plastics, integral to high-end aerospace equipment. Existing research primarily focuses on in-plane strain measurement, limited by the fact that fiber Bragg gratings are mainly sensitive to axial strain. However, out-of-plane strain measurement is equally important for comprehending structural deformation. The birefringence of fiber Bragg gratings shows promise for addressing this problem; yet, the strain transfer relationship between composites and optical fibers, along with the decoupling method for multi-directional strains, remains inadequately explored. This study introduces an innovative method for multi-directional strain measurement in fiber-reinforced plastics using the birefringence of a single-fiber Bragg grating. The strain transfer relationship between composites and embedded optical fibers was derived based on Kollar’s analytical model, leading to the development of a multi-directional strain decoupling methodology. This method was experimentally validated on carbon fiber/polyetherimide laminates under thermo-mechanical loading. Its reliability was confirmed by comparing experimental results and finite element simulations. These findings significantly broaden the application scenarios of fiber Bragg gratings, advancing the in-situ measurement technology crucial for the next generation of high-end aerospace equipment.

## 1. Introduction

Fiber-reinforced plastics (FRPs) are increasingly adopted in high-end aerospace equipment due to their superior strength-to-weight ratio and extraordinary performance [1]. These advanced composite structures are subjected to stringent thermo-mechanical loading conditions during both manufacturing and service, which cause unexpected strain and structural deformations that compromise performance [2]. Consequently, to further save vehicle resources, in-situ strain measurement technologies have become crucial for accurately assessing and managing the deformation behavior of these structures.

Embedded fiber Bragg gratings (FBGs) have been progressively applied for in-situ strain measurement in FRPs due to their small size, lightweight, excellent temperature tolerance, and immunity to electromagnetic interference [3]. Extensive research explored the application of FBGs for full lifecycle strain monitoring in FRPs, advancing from single-point to multi-point measurements [4,5], from simple laminates to complex structures [6,7], and covering manufacturing and verification processes [8,9]. While these studies provided valuable insights into strain distribution within FRPs, they primarily focused on in-plane strain measurement, constrained by the fact that FBGs are mainly sensitive to axial strain. The necessity for out-of-plane strain measurement has become evident, enabling a more comprehensive understanding of structural deformations. For example, the spring-in phenomenon in curved FRP structures is closely related to out-of-plane residual strains [10]. Minakuchi [11] successfully monitored out-of-plane strain by inserting FBGs along the thickness direction of FRP prepregs. However, this method’s applicability was limited due to the fragility of fibers placed through the thickness.

The birefringence effect of FBGs offers a promising solution to the aforementioned issues. Birefringence was previously regarded as a source of strain measurement error, as it caused spectral broadening and even splitting due to non-uniform strain distribution across the cross-section of a sensor [12,13]. In contrast, recent quantitative studies suggested that birefringence could be harnessed to enable transverse strain measurement. Voet et al. [14] successfully applied the birefringence of embedded FBGs to measure transverse strain in FRPs. Nevertheless, the broad linewidth of conventional FBGs (typically around 0.3 nm) posed challenges in observing birefringence [15]. Minakuchi et al. [7] improved birefringence demodulation by measuring the full width at half-maximum (FWHM) of FBGs. Lammens et al. [16] enhanced transverse strain sensitivity through polarization-dependent loss analysis. Ye et al. [17], Wachtarczyk et al. [18], and Gouws et al. [19] used polarization-maintaining FBGs to increase the sensitivity to transverse strain. Chen et al. [12] used the narrow transmission peaks of phase-shifted fiber Bragg grating (PSFBG) to improve transverse strain sensitivity without relying on polarization-dependent loss analysis or polarization-maintaining configurations and successfully decoupled tridirectional strains within a simple isotropic resin plate. However, the decoupling method in reference [12], which depends on the specific strain ratio of optical fibers embedded in isotropic materials, becomes ineffective when applied to FRPs due to their inherent heterogeneity. In summary, two key issues remain unresolved. First, the strain transfer relationship between the host material and the embedded sensor, particularly under thermo-mechanical loading in FRPs, has not been thoroughly investigated. Second, the birefringence of a single sensor alone does not provide sufficient equations to decouple multi-directional strains, leading most studies to focus solely on measuring the difference in transverse strains.

This study proposes a novel approach for measuring multi-directional strains in FRPs by leveraging the birefringence of a single FBG. The strain transfer relationship between composites and embedded optical fibers was established based on Kollar’s analytical model. A multi-directional strain decoupling algorithm was developed for quasi-isotropic FRP laminate subjected to thermo-mechanical loading. This method was experimentally validated on carbon fiber/polyetherimide (CF/PEI) laminates and compared with finite element simulations. The remainder of this paper is organized as follows: Section 2 investigates the mechanism of multi-directional strain measurement, and Section 3 describes the simulation configurations, followed by corresponding experimental setups in Section 4. The results of simulations and experiments are discussed in Section 5. Finally, conclusions are provided in Section 6.

## 2. Mechanism

### 2.1. Sensing Principle

**Figure 1:**
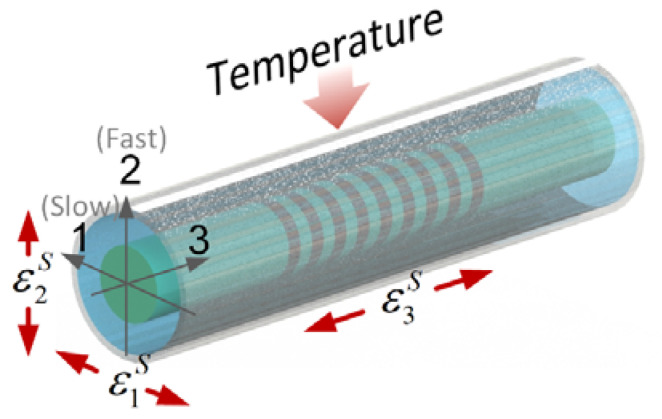
Effects of multi-directional strains and temperature on FBG.

An FBG is manufactured by introducing a periodic grating in the core of an optical fiber. This grating reflects a portion of the incident light around its Bragg wavelength $\lambda_{B}$:

(1) $$\lambda_{B}=2n_{eff}\Lambda ,$$

where $n_{eff}$ denotes the effective refractive index of the grating and Λ represents the grating period. In a single-mode fiber, the propagation of two HE_11_ modes can be resolved into linear polarizations along Axes 1 and 2, as illustrated in Figure 1 [20].

The Bragg wavelength shift $\Delta \lambda_{B,i}$ along Axes 1 and 2 can be described by Equations (2) and (3), respectively [16].

(2) $$\frac{\Delta \lambda_{B,1}}{\lambda_{B}}=\varepsilon_{3}^{S}-\frac{{n_{eff}}^{2}}{2}[p_{11}\varepsilon_{1}^{S}+p_{12}(\varepsilon_{3}^{S}+\varepsilon_{2}^{S})]+\xi \Delta T,$$

(3) $$\frac{\Delta \lambda_{B,2}}{\lambda_{B}}=\varepsilon_{3}^{S}-\frac{{n_{eff}}^{2}}{2}[p_{11}\varepsilon_{2}^{S}+p_{12}(\varepsilon_{3}^{S}+\varepsilon_{1}^{S})]+\xi \Delta T,$$

where $p_{11}$ and $p_{12}$ represent the strain-optic coefficients, ΔT represents the temperature change, and ξ represents the temperature sensitivity. $\varepsilon_{1}^{S}$ and $\varepsilon_{2}^{S}$ are the transverse strains of the optical fiber, and $\varepsilon_{3}^{S}$ is the axial strain. By adding and subtracting Equations (2) and (3), the following relationships are obtained:

(4) $$\frac{\Delta \lambda_{B,1}+\Delta \lambda_{B,2}}{2}=\kappa_{1}\varepsilon_{3}^{S}+\kappa_{2}(\varepsilon_{1}^{S}+\varepsilon_{2}^{S})+\kappa_{3}\Delta T,$$

(5) $$\Delta \lambda_{B,1}-\Delta \lambda_{B,2}=\kappa_{4}(\varepsilon_{1}^{S}-\varepsilon_{2}^{S}),$$

where $\kappa_{i}$ (*i* = 1, 2, 3, 4) is determined by Equation (6), and their values are listed in Table 1:

(6) $$\begin{array}{l} \kappa_{1}=\frac{\lambda_{B}(2-{n_{eff}}^{2}p_{12})}{2}\\ \kappa_{2}=-\frac{\lambda_{B}{n_{eff}}^{2}(p_{11}+p_{12})}{4}\\ \kappa_{3}=\lambda_{B}\xi \\ \kappa_{4}=-\frac{\lambda_{B}{n_{eff}}^{2}(p_{11}-p_{12})}{2} \end{array}\}.$$

Equations (4) and (5) can be represented with the average wavelength shift $\Delta \lambda_{B,A}$ and change in the full-width at half-maximum ΔFWHM, as discussed in our previous work [12]:

(7) $$\Delta \lambda_{B,A}=\kappa_{\varepsilon }\varepsilon_{3}^{S}+\kappa_{T}\Delta T,$$

(8) $$\Delta FWHM=\kappa_{4}(\varepsilon_{1}^{S}-\varepsilon_{2}^{S}),$$

where $\kappa_{\varepsilon }$ and $\kappa_{T}$ denote the axial strain and temperature coefficients, respectively. Therefore, the axial strain $\varepsilon_{3}^{S}$ and transverse strain difference $\left|\varepsilon_{1}^{S}-\varepsilon_{2}^{S}\right|$ can be deduced with Equations (7) and (8), respectively, after compensation for the ΔT. It should be noted that the relative magnitudes of $\varepsilon_{1}^{S}$ and $\varepsilon_{2}^{S}$ cannot be recognized by ΔFWHM alone. Thus, the transverse strain difference herein is represented with $\left|\varepsilon_{1}^{S}-\varepsilon_{2}^{S}\right|$.

### 2.2. Strain Transfer Relationship

The spectral response of FBGs provides strain information only for the optical fiber itself. To accurately monitor strain in FRPs, it is necessary to investigate the strain transfer relationship between the FRP and embedded optical fiber. Kollar’s model is an analytical framework for studying the thermo-mechanical interaction between the host material and embedded optical fiber, bridging the sensor output with the strain and temperature of the host material [21]. Kollar’s model makes the following assumptions: (1) perfect bonding, (2) small deformations, (3) linear elastic materials, and (4) uniform temperature. In Kollar’s model, the strain transfer relationship between the host material and the optical fiber is expressed as follows:

(9) $$\left[\begin{array}{c} \varepsilon_{1}^{S}\\ \varepsilon_{2}^{S}\\ \vdots \\ \varepsilon_{6}^{S}\\ \Delta T \end{array}\right]=TC\left[\begin{array}{c} \varepsilon_{1}^{H}\\ \varepsilon_{2}^{H}\\ \vdots \\ \varepsilon_{6}^{H}\\ \Delta T \end{array}\right]=\left[\begin{array}{ccccc} TC_{11} & TC_{12} & \cdots & TC_{16} & TC_{17}\\ TC_{21} & TC_{22} & \cdots & TC_{26} & TC_{27}\\ \vdots & \vdots & \ddots & \vdots & \vdots \\ TC_{61} & TC_{62} & \cdots & TC_{66} & TC_{67}\\ TC_{71} & TC_{72} & \cdots & TC_{76} & TC_{77} \end{array}\right]\left[\begin{array}{c} \varepsilon_{1}^{H}\\ \varepsilon_{2}^{H}\\ \vdots \\ \varepsilon_{6}^{H}\\ \Delta T \end{array}\right],$$

where TC is the strain transfer matrix. In Equation (9), the coefficients related to the shear strains are zero except for $TC_{44}$, $TC_{55}$, and $TC_{66}$, indicating a one-to-one correspondence between the shear strains of the host material and embedded optical fiber. On the other hand, shear strains have a negligible impact on the refractive index of optical fibers [22]. Therefore, it is assumed that the shear strains of the host material hardly affect the measurement results, allowing Equation (9) to be simplified as follows:

(10) $$\left[\begin{array}{c} \varepsilon_{1}^{S}\\ \varepsilon_{2}^{S}\\ \varepsilon_{3}^{S}\\ \Delta T \end{array}\right]=\left[\begin{array}{cccc} TC_{11} & TC_{12} & TC_{13} & TC_{14}\\ TC_{21} & TC_{22} & TC_{23} & TC_{24}\\ TC_{31} & TC_{32} & TC_{33} & TC_{34}\\ TC_{41} & TC_{42} & TC_{43} & TC_{44} \end{array}\right]\left[\begin{array}{c} \varepsilon_{1}^{H}\\ \varepsilon_{2}^{H}\\ \varepsilon_{3}^{H}\\ \Delta T \end{array}\right],$$

where $TC_{ij}$ (*i*, *j* = 1, 2, 3) are dimensionless quantities, while the units of $TC_{ij}$ (*i* = 1, 2, 3, *j* = 4) are με/°C. Specifically, $TC_{31}$, $TC_{32}$, and $TC_{34}$ are zero, and $TC_{33}$ is always 1, indicating that the strain in the host material and optical fiber are equal in the 3 axial direction; $TC_{41}$, $TC_{42}$, and $TC_{43}$ are zero, and $TC_{44}$ is always 1, corresponding to the assumption of uniform temperature. The remaining coefficients in the TC matrix can be determined using the optical fiber diameter and material properties of the host material and the optical fiber. The diameter of an optical fiber with an FBG is typically 125 μm. This study targets carbon fiber (CF) reinforced polyetherimide (PEI), an advanced thermoplastic composite. The material properties of the constituents and the homogenized properties of the CF/PEI composite are presented in Table 2, along with the properties of commonly used silica optical fibers.

The TC matrix can be calculated using the parameters of the CF/PEI composite and the optical fiber, as shown in Equation (11). The detailed calculation process is not presented here, as has been illustrated in the reference [21].

(11) $$\left[\begin{array}{c} \varepsilon_{1}^{S}\\ \varepsilon_{2}^{S}\\ \varepsilon_{3}^{S}\\ \Delta T \end{array}\right]=\left[\begin{array}{cccc} 0.63996 & 0.15911 & 0.02103 & -8.07728\\ 0.15911 & 0.63996 & 0.02103 & -8.07728\\ 0 & 0 & 1.00000 & 0\\ 0 & 0 & 0 & 1.00000 \end{array}\right]\left[\begin{array}{c} \varepsilon_{1}^{H}\\ \varepsilon_{2}^{H}\\ \varepsilon_{3}^{H}\\ \Delta T \end{array}\right].$$

Since the TC matrix is invertible, Equation (11) also allows the inverse calculation to determine the host material strain from the optical fiber strain.

### 2.3. Decoupling Methodology

**Figure 2:**
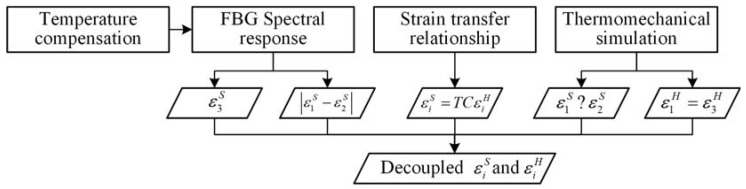
Tridirectional strain decoupling methodology for quasi-isotropic FRP laminate with embedded FBG.

Based on the strain transfer relationship, this study proposes a methodology for decoupling multi-directional strains in quasi-isotropic laminates under thermo-mechanical loading, as shown in Figure 2. First, the axial strain $\varepsilon_{3}^{S}$ and transverse strain difference $\left|\varepsilon_{1}^{S}-\varepsilon_{2}^{S}\right|$ in the optical fiber can be derived from the spectral response of the FBG and temperature compensation, as illustrated in Section 2.1. Since the magnitudes of $\varepsilon_{1}^{S}$ and $\varepsilon_{2}^{S}$ cannot be determined solely from the spectral response of the FBG and Equation (8), the transverse strain difference is expressed as $\left|\varepsilon_{1}^{S}-\varepsilon_{2}^{S}\right|$. Additionally, Equations (7) and (8) provide only two equations involving three variables, which is insufficient for multi-directional strain decoupling.

Second, a strain transfer relationship between the host material and optical fiber is established based on Kollar’s analytical model, enabling the conversion of strains in the CF/PEI composite to those in the embedded optical fiber. This model provides three independent equations but also introduces three new variables, excluding temperature change, which is treated as a known value.

Third, thermo-mechanical finite element (FE) simulation can serve as a powerful tool for determining whether $\left|\varepsilon_{1}^{S}-\varepsilon_{2}^{S}\right|$ equals $\varepsilon_{1}^{S}-\varepsilon_{2}^{S}$ or $\varepsilon_{2}^{S}-\varepsilon_{1}^{S}$. Also, the relationship between $\varepsilon_{i}^{H}$ (*i* = 1, 2, and 3) under specific thermo-mechanical loading can be provided by FE simulation based on the constitutive characteristics of CF/PEI laminates. The relationship among $\varepsilon_{i}^{H}$ can introduce an additional independent equation. In this study, thermo-mechanical experiments on CF/PEI cross-ply laminates are chosen as representative examples. Thus, the following equation can be hypothesized as follows:

(12) $$\varepsilon_{1}^{H}=\varepsilon_{3}^{H},$$

which will be validated through the subsequent thermo-mechanical simulation. Finally, by substituting the measured optical fiber strains into the six independent equations, decoupling of the tridirectional strains of the optical fiber and the FRP can be achieved.

## 3. Simulation

### 3.1. Finite Element Model

**Figure 3:**
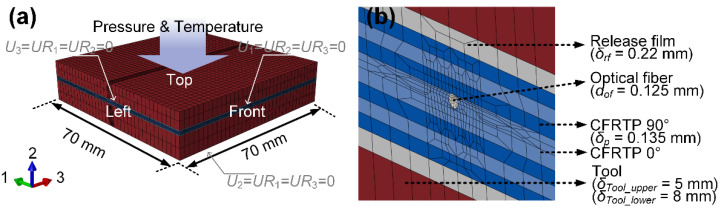
FE-model of CF/PEI composite with embedded PSFBG: (**a**) global view, (**b**) enlarged view.

An FE simulation was conducted to verify the strain decoupling method and compare it with the experiments. The FE model was not symmetric along the thickness due to the presence of the optical fiber. Therefore, a 1/4 model of the CF/PEI laminate with the embedded optical fiber was developed, matching the subsequent experimental geometry, as shown in Figure 3. The red regions represent the upper and lower steel mold plates with dimensions of 70 × 70 × 6 mm^3^ and 70 × 70 × 8 mm^3^ (length × width × height), respectively. The white regions indicate the release film with dimensions of 70 × 70 × 0.22 mm^3^. The blue region represents the CF/PEI laminate, with dimensions of 70 × 70 × 1.08 mm^3^. The thickness is the average of multiple measurements of the manufactured CF/PEI laminate. The material orientation matches the layup [(90/0)_2_]_s_ used in the experiments. The white cylinder in the center represents the optical fiber, with a diameter of 125 μm. The coating is excluded because the PI coating strain does not affect the strain transfer relationship between host materials and embedded optical fibers [12]. The material properties are listed in Table 2. The FE model is meshed by C3D20R elements with a global mesh size of 1 mm, and the surrounding of the optical fiber is fine-meshed with a minimum size of 0.025 mm, resulting in a total node number of 350,346.

### 3.2. Boundary Condition

The left surface is fixed along axis 3 with rotations around axes 1 and 3 constrained. The front surface is fixed along axis 1 with rotations around axes 2 and 3 constrained. The bottom surface is fixed along axis 2. The temperature of the whole FE model is set according to the thermocouple measurement from the experiment. The pressure on the top surface is set to 2 MPa. The analysis increment is set as 20 s.

## 4. Experiment

### 4.1. Sensors

**Figure 4:**
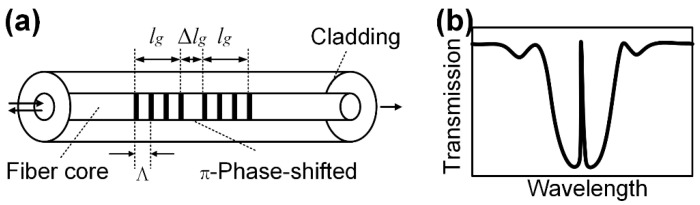
Characteristics of PSFBG: (**a**) structure, and (**b**) transmission spectrum.

This study utilized a specially designed FBG, PSFBG (1550 nm, TONGWEI, Chengdu, China), which can improve the sensitivity of transverse strain [12]. The PSFBG illustrated in Figure 4a features a grating length of $2l_{g}+\Delta l_{g}$, a grating period of Λ, and a π phase shift at the middle of its grating, making it possess an extremely narrow transmission peak (1~30 pm), as shown in Figure 4b. The presence of the narrow transmission peak results in a short effective grating length (~1 mm), giving PSFBGs higher spatial resolution compared to conventional FBGs. This makes them less prone to spectral distortion caused by grating chirping when embedded in composite under complex strain loads. Additionally, the narrow transmission peak can easily demonstrate birefringence without necessitating a polarization-maintaining optical path or polarization-dependent loss analysis. The diameters of the core, cladding, and polyimide coating of the PSFBG are 9, 125, and 155 μm, respectively.

### 4.2. Experiment System

**Figure 5:**
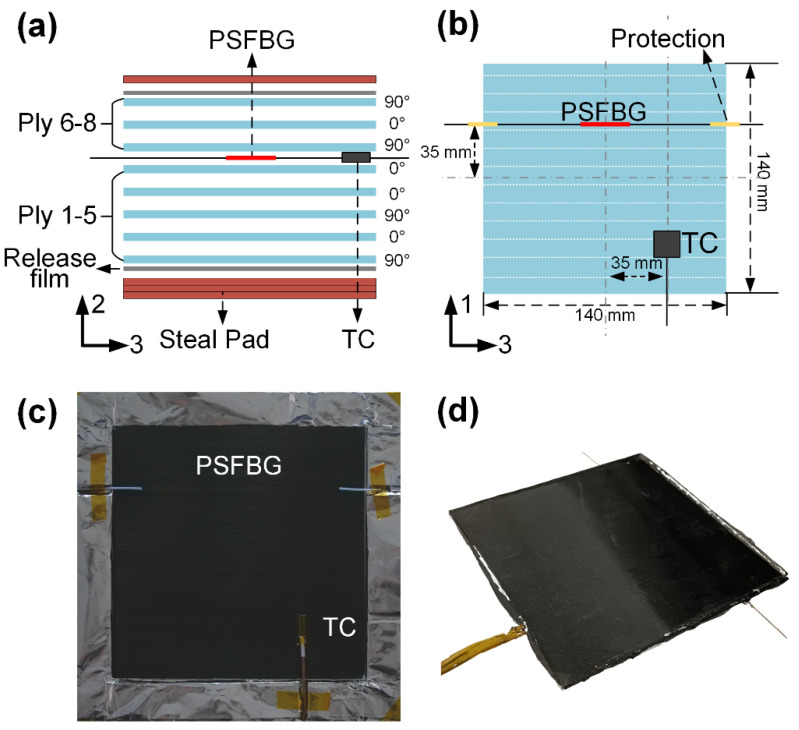
CF/PEI prepreg and sensor setup: (**a**) main view, (**b**) top view, (**c**) photograph, and (**d**) manufactured CF/PEI laminate with embedded sensors.

This study experimentally applies the multi-directional strain measurement method to monitor the thermo-mechanical experiments on an advanced thermoplastic composite, specifically stacked by unidirectional composite prepregs (Cetex^®^ TC1000 PEI AS4, TORAY, Tokyo, Japan) comprising CF (AS4, HEXTOW, Stamford, CT, USA) and PEI resin. PEI possess glassy, leathery, rubbery, and viscous states, which are divided by the glass transition temperature $T_{g}$ of 210 °C, the rubbery plateau beginning temperature $T_{p}$ of 227 °C, and the liquid-liquid transition (rubbery-viscous) temperature $T_{ll}$ of 250 °C. This study focuses the multi-directional strain measurement under $T_{g}$. Eight prepreg plies with dimensions of 140 × 140 × 0.135 mm^3^ (L × W × T) were laid according to the sequence [(90/0)_2_]_s_, as depicted in Figure 5a. For the given CF/PEI laminates, the in-plane properties can be considered uniform. This means that when the composite is subjected to uniformly distributed out-of-plane loads and a homogeneous in-plane temperature field, the strains in both in-plane directions should be identical. A PSFBG was embedded between the 5th and 6th plies and aligned in the 0° direction, as illustrated in Figure 5a,b. A thermocouple (TC) (C060-K, CHINO) was also prepared for temperature compensation. Figure 5c shows the photograph taken when laying the sensors on the 5th ply.

**Figure 6:**
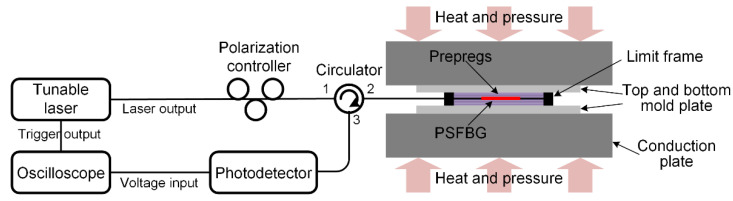
Experiment system including interrogation system and hot press.

The interrogation system must have a spectral resolution matching the FWHM to fully exploit the advantage of the extremely narrow transmission peak of the PSFBG for high-precision strain measurement. Therefore, the interrogation system requires a spectral resolution of less than 1 pm, which conventional interrogation instruments cannot achieve. A high-resolution interrogation system capable of resolving $\Delta \lambda_{B,A}$ and ΔFWHM was developed [12]. The main components of the interrogation system shown in Figure 6 are as follows: (1) Tunable laser (TSL-710, SANTEC, Aichi, Japan): delivers precise, high-speed swept laser with a wavelength range of 1480–1640 nm, linewidth less than 100 kHz, wavelength resolution of 0.1 pm, and sweep speed up to 100 nm/s; (2) Polarization controller (MPC320, THORLABS, Newton, New Jersey, USA): manages the polarization state of the input light with three rotating paddles, each capable of 160° rotation and a minimum step size of 0.12°; (3) Circulator: directs optical signals; (4) Photodetector (PDA10CS2, THORLABS, New Jersey, USA): converts optical signals to electrical signals with a built-in low-noise trans-impedance amplifier. It detects wavelengths from 900 to 1700 nm with a bandwidth of DC to 13 MHz; (5) Oscilloscope (DSOX2004A, KEYSIGHT, Colorado Springs, CO, USA): converts and acquires digital signals from analog inputs, with a bandwidth of 70 MHz and a maximum sampling rate of 2 GSa/s. 

The interrogation system operates as follows: (1) The tunable laser emits narrowband light with a sweeping rate of 100 nm/s, simultaneously providing a trigger signal to the oscilloscope; (2) The polarization controller adjusts the polarization state of the output light, which is then directed into the circulator; (3) The circulator routes the reflected light from the PSFBG to the photodetector, where it is converted into an electrical signal and sent to the oscilloscope; (4) The oscilloscope begins recording data upon receiving the trigger signal, capturing the electrical signal measured by the photodetector overtime during the wavelength sweep; (5) The wavelength is deduced by multiplying the time coordinate by the sweep rate, while the reflectivity is obtained by dividing the voltage value by that recorded when a full-reflecting mirror is connected to channel two of the circulator. In this study, reflection spectra were converted into transmission spectra for easy description and analysis since the spectral feature to be demodulated is the narrow transmission peak of the PSFBG. This high-precision interrogation system can achieve a spectral sampling rate as fast as 0.1 s and a spectral resolution of 0.1 pm. The recorded spectra allow for precise determination of the Bragg wavelength and FWHM of PSFBGs.

Laminated prepregs with the sensors were placed in a hot press (HBSCR-50T/400AV, HUABO, Qingdao, China), as shown in Figure 6. Heat and pressure were applied through two conduction plates. The top and bottom mold plates and limit frame were used to limit the movement of the CF and PEI. A cooling process was set for strain measurement, which involved the temperature from 210 to 60 °C. Two sets of experiments were conducted with different pressures of 1 MPa and 2 MPa.

## 5. Results and Discussion

### 5.1. Simulation Results

**Figure 7:**
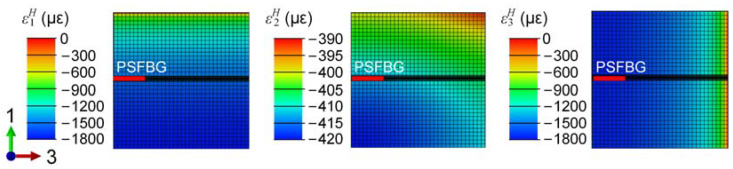
Strain distribution in ply 5 containing PSFBG.

Figure 7 shows the simulated strain distribution of the ply 5 in the CF/PEI laminate. The PSFBG is embedded in this ply, with its position indicated by the red line in Figure 7. The out-of-plane strain $\varepsilon_{2}^{H}$ is larger than the in-plane strains $\varepsilon_{1}^{H}$ and $\varepsilon_{3}^{H}$ due to the tool-part interaction. The CTE of the steel mold (18.4 × 10^−6^ °C^−1^) is greater than that of the composite (see Table 2). Thus, the thermally induced contraction of the steel plates is more significant and is transferred to the laminate. The similarity between $\varepsilon_{1}^{H}$ and $\varepsilon_{3}^{H}$ in Figure 7 confirms the hypothesis that the in-plane strains of the CF/PEI composite with [(90/0)_2_]_s_ lay-up are equal during the cooling process, providing an important new equation for multi-directional strain decoupling, as indicated by Equation (12).

**Figure 8:**
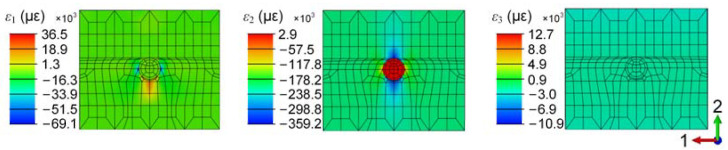
Strain distribution around PSFBG.

Figure 8 presents the simulation results of the micro-scale around the optical fiber. The transverse strains $\varepsilon_{1}^{S}$ and $\varepsilon_{2}^{S}$ in the optical fiber show discrepancies compared to the corresponding strains in the composite $\varepsilon_{1}^{H}$ and $\varepsilon_{2}^{H}$. Additionally, strain gradient within approximately 0.25 mm around the optical fiber confirms the strain transfer relationship between the host material and the embedded optical fiber. The axial strain $\varepsilon_{3}^{S}$ in the optical fiber is nearly equal to that in the composite, consistent with the conclusion in Section 2.2 that the axial strain transfer coefficient $TC_{33}$ is always 1. The values of $\varepsilon_{1}^{S}$, $\varepsilon_{2}^{S}$, and $\varepsilon_{3}^{S}$ in the optical fiber are 123, 514, and −1809 με, respectively, indicating that $\varepsilon_{2}^{S}$ is greater than $\varepsilon_{1}^{S}$. This provides critical information for the tri-directional strain decoupling, suggesting that the transverse strain monitoring results in the experiment can be determined as follows:

(13) $$\left|\varepsilon_{1}^{S}-\varepsilon_{2}^{S}\right|=\varepsilon_{2}^{S}-\varepsilon_{1}^{S},$$

### 5.2. Strains Derived from Spectral Response

**Figure 9:**
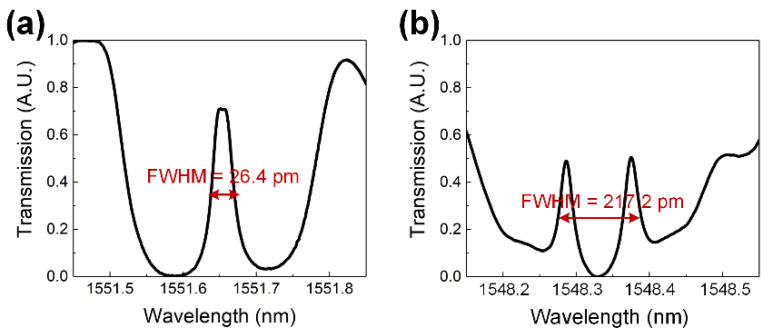
Spectra of embedded PSFBG: (**a**) at 210 °C, (**b**) at 60 °C.

Figure 9 shows the transmission spectra of the PSFBG at 210 °C and 60 °C. At the beginning of the experiment, the initial transmission spectrum of the PSFBG resembles a typical Lorentzian curve [23], with a slight peak separation, indicating the presence of initial birefringence. In cases where no obvious peak separation appears, it is assumed that $\Delta \lambda_{B,1}$ and $\Delta \lambda_{B,2}$ are equivalent. The narrow transmission peak has a transmission of 0.74, an average Bragg wavelength of 1551.84 nm, and an FWHM of 25.4 pm. At 60 °C, the narrow transmission peaks of the fast and slow axes show significant separation; thus, the average Bragg wavelength $\lambda_{B,A}$ is calculated as $\left(\lambda_{B,1}-\lambda_{B,2}\right)/2$. The average Bragg wavelength of the narrow transmission peak shifts to a shorter wavelength of 1548.30 nm, with the FWHM broadening to 217.2 pm.

**Figure 10:**
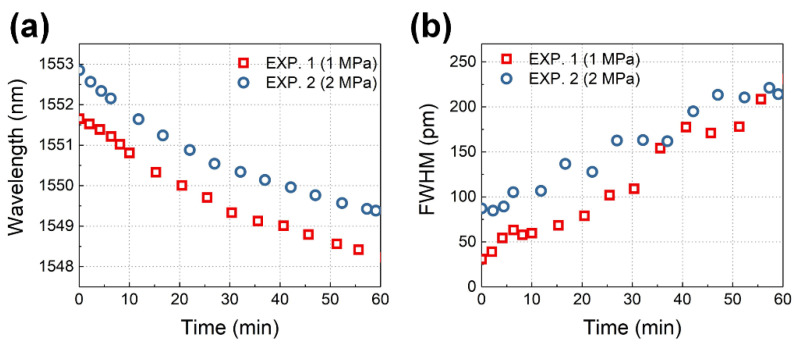
Spectral characteristics: (**a**) average Bragg wavelength, and (**b**) FWHM.

The Bragg wavelength and FWHM curves during the experiments are shown in Figure 10 by analyzing the PSFBG transmission spectrum. The Bragg wavelength shift in Figure 10a is caused by the axial strain $\varepsilon_{3}^{S}$ in the optical fiber and temperature change ΔT according to Equation (7). The FWHM in Figure 10b is induced by the transverse strain difference $\left|\varepsilon_{1}^{S}-\varepsilon_{2}^{S}\right|$ in the optical fiber according to Equation (8).

**Figure 11:**
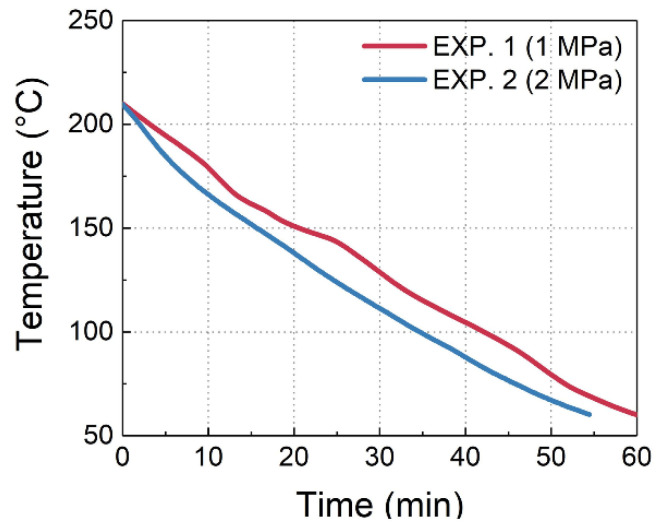
Temperature curves.

**Figure 12:**
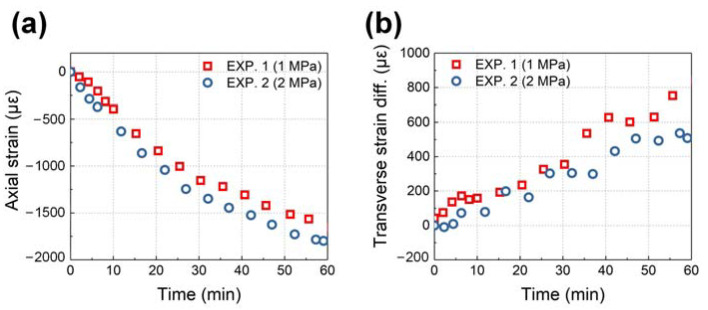
Coupled strains derived from the spectral response of PSFBG: (**a**) axial strain and (**b**) transverse strain difference.

By substituting the temperature monitoring results in Figure 11 into Equation (7), the axial strain $\varepsilon_{3}^{S}$ and transverse strain difference $\left|\varepsilon_{1}^{S}-\varepsilon_{2}^{S}\right|$ of the PSFBG can be calculated using Equations (7) and (8) and plotted in Figure 12.

### 5.3. Tri-Directional Strains

**Figure 13:**
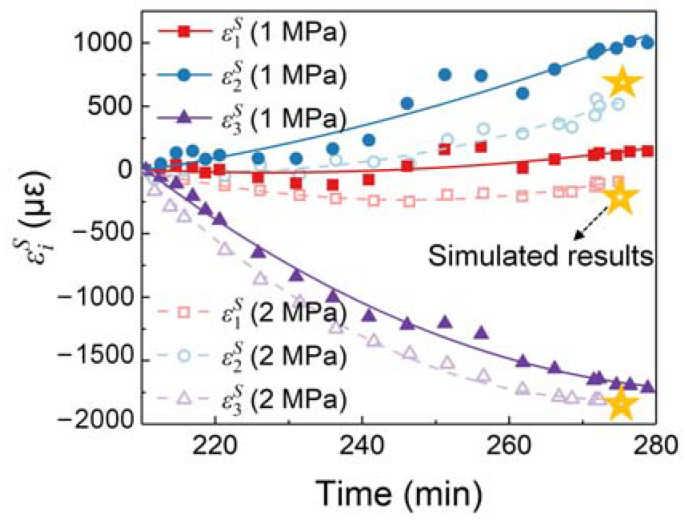
Tri-directional strains in optical fiber.

By substituting the strains derived from the spectral response at each moment into the methodology shown in Figure 2, the tridirectional strains of the PSFBG can be decoupled and plotted in Figure 13. The tridirectional strains exhibit similar characteristics in both sets of experiments: the absolute value of $\varepsilon_{1}^{S}$ is minimal and remains nearly zero; $\varepsilon_{2}^{S}$ is tensile strain and increases monotonically over time; $\varepsilon_{3}^{S}$ is compressive and decreases monotonically over time. $\varepsilon_{1}^{S}$ and $\varepsilon_{2}^{S}$ are influenced by the Poisson effect induced by $\varepsilon_{3}^{S}$. Therefore, although the CF/PEI composite theoretically contracts during the cooling process, the embedded optical fiber may not contract in the transverse direction.

There are some differences in the tridirectional strains between the two experiments under different pressures. The strains in EXP. 2 are generally smaller than those in EXP. 1. Specifically, the difference of $\varepsilon_{2}^{S}$ agrees with the experimental condition; for example, the embedded optical fiber in the composite experiences greater compression under higher pressure, resulting in reduced strain values. The yellow pentagram in Figure 13 represents the simulation results corresponding to EXP. 2. The differences between the experimental and simulation results for $\varepsilon_{1}^{S}$, $\varepsilon_{2}^{S}$, and $\varepsilon_{3}^{S}$ are 102, 115, and 44 με, respectively, demonstrating the consistency between the experimental and simulation results.

**Figure 14:**
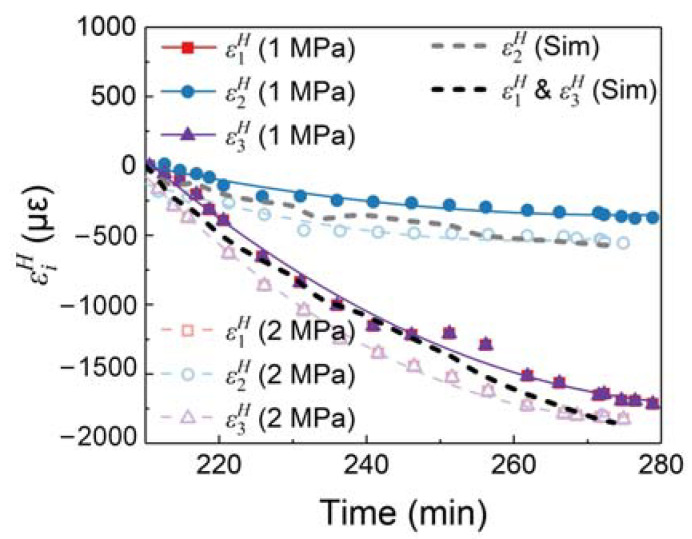
Tridirectional strains in CF/PEI laminate.

The tridirectional strain measurements for the CF/PEI laminate are calculated and presented in Figure 14. Given the precondition for tridirectional strain, decoupling is $\varepsilon_{1}^{H}=\varepsilon_{3}^{H}$, and the in-plane strains $\varepsilon_{1}^{H}$ and $\varepsilon_{3}^{H}$ are equal in both EXP. 1 and EXP. 2. Furthermore, all tridirectional strains in the composite are compressive and decrease monotonically over time.

The in-plane strains $\varepsilon_{1}^{H}$ and $\varepsilon_{3}^{H}$ are smaller than the out-of-plane strain $\varepsilon_{2}^{H}$, indicating that the CF/PEI laminate contracts more in-plane than out-of-plane direction. If no constraint is applied on the CF/PEI laminate, in-plane contraction should be less than out-of-plane contraction due to the reinforcement of CFs. However, the in-plane contraction was influenced by the tool-part interaction in the experiments. During cooling, the significant thermal contraction of the mold was transferred to the composite through the release film. The out-of-plane strain $\varepsilon_{2}^{H}$ shows significant differences between EXP. 1 and EXP. 2 due to the different pressures applied. The results indicate that the higher the pressure, the more significant the compression in the 2-axial direction, which aligns with the fact.

The black dashed lines in Figure 14 represent the simulation results corresponding to EXP. 2. The experimental tridirectional strain measurements based on PSFBG show good agreement with the simulation results, with average differences of 97, 97, and 52 με for $\varepsilon_{1}^{H}$, $\varepsilon_{2}^{H}$, and $\varepsilon_{3}^{H}$, respectively. At the end of the cooling process, the experimental deviations from the simulation were 5.3%, 5.3%, and 9.4%, respectively. This confirms the reliability of the tridirectional strain measurement of CF/PEI composites using the birefringence of embedded FBGs under thermo-mechanical loading.

## 6. Conclusions

This study primarily focused on the development and validation of a multi-directional strain measurement method using FBG embedded in CF/PEI composites. The strain transfer relationship between composites and embedded optical fibers was established based on Kollar’s analytical model. Combined with the simulation results, a multi-directional strain decoupling algorithm was developed for quasi-isotropic FRP laminate subjected to specific thermo-mechanical loading conditions. An experimental investigation was conducted to apply PSFBGs in CF/PEI composites under thermo-mechanical loading. The decoupling and measurement of tridirectional strains were successfully achieved using PSFBGs embedded within the CF/PEI cross-ply laminate. The experiments demonstrated that the PSFBGs exhibited distinct strain behaviors in different directions, with the axial strain $\varepsilon_{3}^{S}$ being nearly identical to the $\varepsilon_{3}^{H}$ within the composite. Notable differences were observed in the transverse strains $\varepsilon_{1}^{S}$ and $\varepsilon_{2}^{S}$, confirming the strain transfer relationship between the composite and the optical fiber. In the measurement results for the CF/PEI composite, all tridirectional strains were compressive and decreased monotonically over time. The in-plane strains $\varepsilon_{1}^{H}$ and $\varepsilon_{3}^{H}$ were smaller than the out-of-plane strain $\varepsilon_{2}^{H}$, indicating a more significant in-plane shrinkage due to the tool-part interaction during the cooling process. Comparing the experimental results with the FE simulation, the tridirectional strain measurements of the CF/PEI composite showed good agreement, with average differences of 97 με, 97 με, and 52 με for $\varepsilon_{1}^{H}$, $\varepsilon_{2}^{H}$, and $\varepsilon_{3}^{H}$, respectively. At the end of the cooling process, the experimental deviations from the FE simulation were 5.3%, 5.3%, and 9.4%, respectively, demonstrating the reliability of this newly proposed method for multi-directional strain measurement of CF/PEI composites. The strain transfer relationship and decoupling methodology are suitable not only for CF/PEI composites with embedded PSFBGs but also for other kinds of FRPs and FBG-based sensors.

This research successfully addressed critical challenges in multi-directional strain measurement in FRPs, forming a more comprehensive technique for strain measurement using birefringence of embedded FBGs. These findings will contribute to the development of in-situ strain measurement technologies and help to improve the quality and safety of high-end aerospace equipment.

## Figures and Tables

**Table 1:** Values.

Parameter	Value
$\kappa_{1}$ (pm/με)	1.106
$\kappa_{2}$ (pm/με)	−0.321
$\kappa_{3}$ (pm/°C)	10.2
$\kappa_{4}$ (pm/με)	0.245

**Table 2:** Properties of CF, PEI, homogenized composite, and silica optical fiber.

Parameter	CF	PEI	Composite	Optical Fiber
$\mathrm{Young}’s\;\mathrm{modulus}\;E_{11}=E_{22}$ (MPa)	17,200	3100	9608	70,000
$\mathrm{Young}’s\;\mathrm{modulus}\;E_{33}$ (MPa)	228,000		135,708	
$\mathrm{Poisson}’s\;\mathrm{ratio}\;v_{13}=v_{23}$	0.2	0.36	0.25	0.17
$\mathrm{Poisson}’s\;\mathrm{ratio}\;v_{12}$	0.5		0.46	
$\mathrm{Shear}\;\mathrm{modulus}\;G_{13}=G_{23}$ (MPa)	27,600	1140	5036	30,000
$\mathrm{Shear}\;\mathrm{modulus}\;G_{12}$ (MPa)	5730		2738	
$\mathrm{CTE}\;\alpha_{11}=\alpha_{22}$ (°C^−1^)	7.2 × 10^−6^	55.8 × 10^−6^	12.7 × 10^−6^	5.5 × 10^−7^
$\mathrm{CTE}\;\alpha_{33}$ (°C^−1^)	−0.9 × 10^−6^		−0.667 × 10^−6^	

## Data Availability

The raw data supporting the conclusions of this article will be made available by the authors on request.
